# ‘“*For a better life …*“ A study on migration and health in Nicaragua’

**DOI:** 10.1080/16549716.2018.1428467

**Published:** 2018-07-05

**Authors:** Cecilia Gustafsson

**Affiliations:** Centre for Demographic and Ageing Research (CEDAR)/Department of Geography and Economic History, Umeå University, Umeå, Sweden

**Keywords:** Migration and health, remittances, mobile livelihoods and translocal geographies, Nicaragua

## Abstract

**Background**: Nicaraguans have migrated internally and internationally for centuries due to economic, political and sociocultural factors. Deficiencies in the country’s health care system have produced inequities in people’s access to health care and medicines. Remittances have become an important source of income, partly invested in health.

**Objectives**: The overall aim of the study was to analyse migration–health relations in contemporary Nicaragua within a broader context of socio-economic transformations.

**Methods**: The study uses a mixed-methods approach, combining qualitative interview data and quantitative survey data.

**Results**: The findings show that migration is commonly practised as a strategy for making a living and is related to the struggle for a better life. Health concerns are indirectly embedded in people’s mobile livelihoods, but also directly influence migration motives. Furthermore, migration involves both advantages and disadvantages for health. Physical and sexual violence can come to an end for migrating women, health care and medicine can become more accessible for internal migrants, and vulnerabilities caused by environmental disasters can be avoided by moving. Moreover, remittances can improve people’s everyday life and health. Yet migration can also be a stressful and health-damaging event. International migrants, particularly the undocumented, can have problems accessing health care, and also experience much danger at border crossings. Transnational families can suffer emotionally as well as physically due to separation. Findings from the survey show that family members of migrants do not rate their physical health as good as often as non-migrating families.

**Conclusions**: The Nicaraguan population is not guaranteed its social rights of citizenship. This results in mobile livelihoods and the need for translocal social support (e.g. remittances). Migration can have both positive and negative effects on health for migrants and their family members; geographical distance and social differences are key to the outcome.

## Background

This article is a review of the PhD thesis ‘“*For a better life …”* A Study on Migration and Health in Nicaragua’ authored by Cecilia Gustafsson [1]. The literature on the linkages between migration and health provides inconclusive answers regarding the effects of migration and health, and vice versa, due to the diversity of migration patterns, migrant groups and research designs [2,3]. The existing research on migration and health in Latin America is limited and mostly un-contextualized [4]. Therefore, the ambition of this thesis is to critically explore and analyse the migration–health nexus in the case of Nicaragua as embedded in the broader local–global context by asking: *In what ways are Nicaraguan migration patterns and health trends related to past and present socio-economic transformations and to social differentiations?*

**Figure 1.**
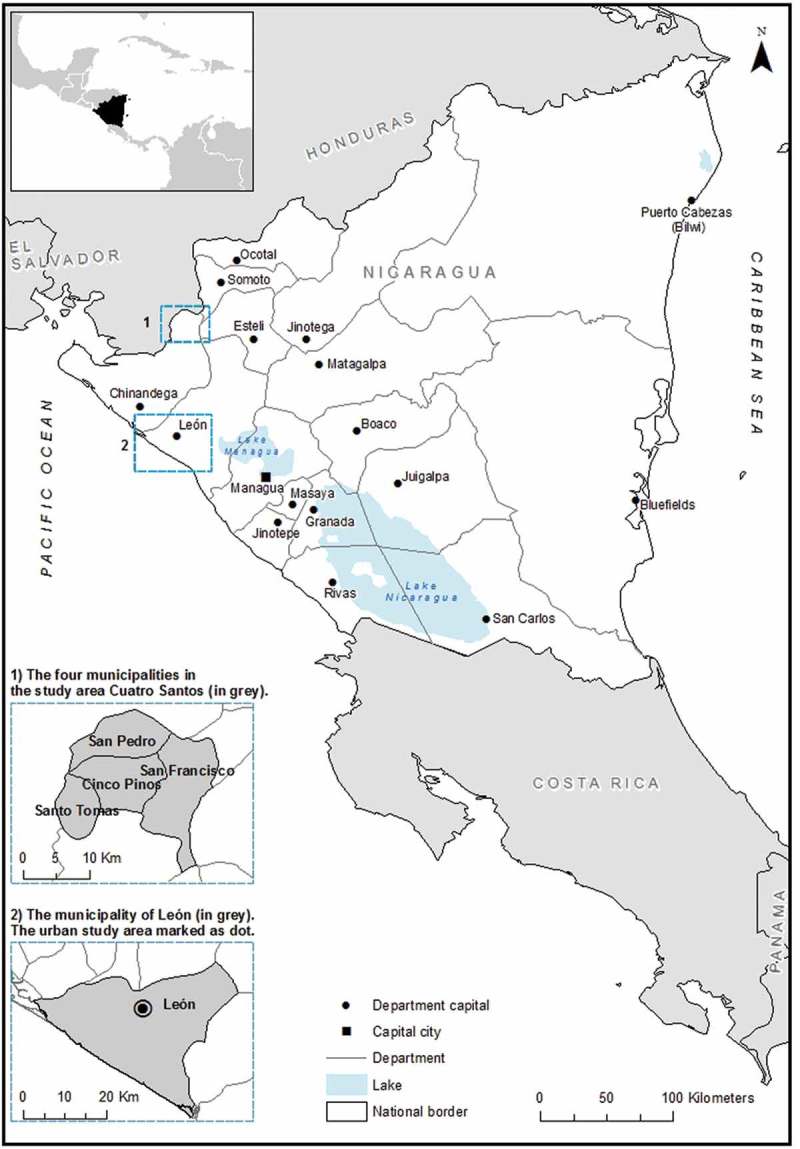
Map of the study settings.

With inspiration from health geography [5], for example, an integrative and social view of health is applied in the thesis. Health is understood as composed of biological, psychological and social factors, in accordance with the biopsychosocial model of health [6]. The importance of the local context, and of relations between individuals and the local and the wider contexts, for understanding health is also central in the thesis [7]. From a critical standpoint, the importance of power relations in producing and reproducing inequalities in health is also stressed. Moreover, as argued in mind/body medicine [8], physical health and mental health are seen as tightly connected.

As there are bidirectional connections between migration and health [9], the thesis scrutinizes both the diverse impacts of migration on health, and the varying effects of health on migration; for example, through the questions: *How are health concerns integrated into migration decisions? How are health issues related to remittance patterns?* Based on relational understandings, migration is understood as a process that binds together everyday lives across spaces, places and scales, thus creating ‘translocal geographies’ [10]. Furthermore, based on previously developed frameworks for analysing migration–health relations [11,12], health issues are investigated in relation to the whole process of migration. Thus, the thesis scrutinizes migration–health relations in places of origin, during travel, at the destination and after return, including the situation and consequences for both migrants and their families. I therefore also ask: *How do migration events affect the migrants, the family members of migrants, and family relations?*

The findings of the thesis are grouped into three overarching themes. The first theme (‘Mobile Livelihoods and Health Dynamics’) investigates migration patterns and migration motives, and how health issues are embedded in people’s strategies for making a living. The second theme (‘Health on the Move’) scrutinizes how migrants’ health is affected during travel, at the destination and after return to the place of origin. Lastly, the third theme (‘Health and Coping in Divided Families’) addresses how migration affects migrant families and how people cope with the separation from family members.

The study takes place at a time when the former revolutionary Sandinistas had regained power after decades of conservative rule and neo-liberal policies. Nicaragua was characterized by, for example, widespread poverty, low income and educational levels, little formal employment and a large informal sector, high degrees of underemployment and child labour, and large gender as well as geographical socio-economic differences [13–18]. Maternal and child mortality [19], as well as violence against women [20–23] were particularly problematic health issues. Moreover, there were substantial deficiencies in the country’s health care system, producing socio-economic and geographical inequities in people’s access to health care and medicines. Households’ out-of-pocket expenditures were about 50% of all spending on health care (medicines constituted the largest part of the expenditures). Individuals with higher incomes and educational levels, and with health insurance, more often received treatment. The richer tended to use services of a higher quality (e.g. private clinics), while the poorer more commonly used public facilities that were free of charge but often of poor quality. Most of the health care services were located on the Pacific coast, with the result that access to care, particularly more specialized care, were limited in sparsely populated areas (e.g. the Caribbean region), and there were also large deficiencies of the services in these areas [24–26].

The Nicaraguan population has migrated for economic, political and sociocultural reasons for centuries. In earlier time periods, people mostly moved within the country, from rural to urban areas, or within the closest regions, often in connection to agricultural seasonal labour, in the search for jobs. During the revolutionary war many took refuge in neighbouring countries, particularly Costa Rica, or the US. Due to the socio-economic situation, which became particularly harsh after the 1990s, migration has become an increasingly international process as more and more Nicaraguans have emigrated in search of employment. Today, about 10–20% of the population resides abroad, primarily in Costa Rica and the US [14,27–30]. The inflow of remittances has increased over time, and they now constitute about 10% of Nicaragua’s gross domestic product [18].

## Methods

Following a mixed-methods research design, the study combines qualitative interview data and quantitative survey data. A combination of the convergent parallel design and the exploratory sequential design [31] was used. The qualitative and quantitative studies were given equal priority and were conducted concurrently. Moreover, some of the questions in the survey stemmed from the results of the qualitative interviews, and some of the interviewees were selected by use of the HDSS (Health and Demographic Surveillance Systems; see below) as well as in relation to the survey. The findings were integrated during the analysis and in the presentation of the results. The research was conducted from a pragmatic standpoint [32], thus destressing the paradigmatic divides between qualitative and quantitative methods, and focusing on the complementarity of the data in order to produce a more complete understanding of the phenomena under study.

The empirical material was gathered through fieldwork in the town of León and the predominantly rural area Cuatro Santos (Figure 1), between the years 2006 and 2008 (with a follow-up visit in 2013). The total time spent in the field was approximately six months.

### The in-depth interviews

Seventeen interviews, between 30 and 120 minutes in length, were conducted (with 5 men and 12 women), whereof 15 were recorded and transcribed. A snowball sampling method was used to reach 12 of the interviewees, while the other 5 were selected in connection to the survey due to their characteristics. The interviewees were aged between 22 and 60 years, and had various educational backgrounds and working experience. They originated in both rural and urban areas, and were presently living either in their place of birth, in another town or abroad. They had experience of internal and/or international migration (both legal and/or irregular migration), either as migrants themselves and/or as family members of a migrant. Following the grounded theory approach [33] the number of interviews undertaken was determined by when the point of saturation was reached. This is, moreover, the reason why more women than men were interviewed.

The majority of the interviews were biographical in-depth interviews [34–36] aiming at exploring and situating the connections between migration and health within the interviewees’ life histories. The analysis was first performed by use of constructivist grounded theory [33], which consisted of coding the interview transcripts by hand, and by use of the computer software MAXQDA10, and grouping the codes into themes that integrated a substantial number of the codes. It was then supplemented with the biographical approach, through which the results could be situated in time, as part of the individual’s life course (see [1], pp. 54–67 for further details on the interview study). The end product of this process is seen in the themes and narratives of the thesis’ empirical chapters, of which a condensed version is presented in this article.

### The two-step survey study

A survey (n = 1,383) was conducted in two steps within the frames of the HDSS in León and Cuatro Santos. These surveillance systems regularly gather population-based data in order to monitor demographic processes in the population, and to conduct research. The data include information on, for example, socio-economic characteristics and demographic events. The HDSS in León dates back to the 1990s, and has continuously been updated and expanded [19,37,38]. At the time of this study the HDSS-León included about 56,000 individuals in 13,000 households (31% of the population). The HDSS in Cuatro Santos was initiated in 2004 and covers all residents in the area, which in 2007 amounted to over 24,000 individuals in 4800 households [39].

In the first step of the survey (2007), the individuals who would constitute the sample in the second step (2008) were singled out through questions on health status. All households in Cuatro Santos were asked these questions, whereas in León every fifth household (2500) in the urban sector was selected, due to limited resources. The study population for the 2008 survey consisted of 40,313 individuals (León: 13,171; Cuatro Santos: 27,142). In the sample process the collected data on health status was first combined with migration data from the HDSS. Then nine different sample groups were created according to migration experience and individual health status (see Table 1). Individuals younger than 17 years were excluded. The sample amounted to 1718 individuals, whereof 1383 completed the survey (León: 572; Cuatro Santos: 811). The response rate was overall 81%.

**Table 1.** The sample groups in the survey.

1a. Non-mover, healthy	2a. Left-behind, healthy	3a. In-migrant, healthy
1b. Non-mover, chronically ill	2b. Left-behind, chronically ill	3b. In-migrant, chronically ill
1c. Non-mover, other illnesses	2c. Left-behind, other illnesses	3c. In-migrant, other illnesses

Notes:

Non-mover: person who still lived in his/her place of birth; no migration history in the family

Left-behind: family member of out-migrant; no personal migration history

In-migrant: person who had moved into the household from another place, no record of migration

Healthy: person who reported no health problems

Chronically ill: person who reported at least one chronic health problem

Other illnesses: person who reported at least one acute/other health problem (but not chronic)

The questionnaire included questions on, for example, migration events, self-rated health, use of health services and medicine, and support within social networks. Questionnaires were completed face to face between trained fieldworkers and the respondents. In the first step of the survey, the respondent was often the head of household. (This is the usual procedure in HDSS rounds; see [37] for more details.) In the second step, the respondent was the sampled individual. The information from the questionnaires was after quality control entered in databases by the collaborating partners, and sent via email to the author.

The main methods for analysing the survey data were descriptive statistics and binary logistic regression analysis. The primary aim of the regressions was to explore associations between migration categories, socio-economic characteristics and health indicators. In the regressions I investigated, for instance, whether associations could be found between health status (using e.g. self-rated physical health as dependent variable) and individuals with different migration backgrounds (based on the categorization in the sample), as well as with different socio-economic status (based on e.g. educational levels, occupation and poverty levels). I also looked at what characterized certain groups in the material – for example, remittance-receivers – to see whether there were any associations between those who received remittances and indicators of health (e.g. self-reported illnesses). As some sample groups were larger than others they were weighted according to their original sizes in the analyses, by use of the software IBM SPSS Complex Samples, in order not to skew the results.

## Results

The presentation of the findings from the empirical material is structured thematically around the main findings of the interview study. The quantitative study illustrates the magnitude of certain aspects, and investigates specific associations between migration and health. Due to lack of space, reference to only some of the quantitative results is provided in this article (see chapters 5–7 in [1] for the complete presentation of results).

### Mobile livelihoods and health dynamics

The study shows that migration was common in the study setting. Even though not all study participants had personal experience of migration, migrant networks were salient features, although they varied in extent and character. Of noticeable importance, over two-thirds (68%) in the survey had family members residing abroad. The observed migration patterns may contribute to further transnational migration, when seen in light of the research on migration dynamics [40–44]. Furthermore, the study confirms previous evidence [45] of extensive irregular migration from Nicaragua: in the survey over a third (36%) had family members abroad who were undocumented.

The study findings indicate a diverse and complex pattern, and a blurring of migration motives. In the survey two-thirds (68%) expressed economic motives for wanting to move (e.g. unemployment, poor income, to improve work situation). Over a third (35%) mentioned social reasons and a few mentioned education (5%) and health (2%). (These findings are, however, based on a relatively small number of observations as only 11% of the respondents had thought of moving and thereby replied to the question concerning motives for moving.) Even though economic factors were accentuated – both in the survey and in the interviews – other reasons (e.g. social relations, education and health) were also expressed. The interviews showed that other reasons were often intertwined with the economic factors. As previous research shows, migrants’ decision-making is based on a variety of considerations [46–48], and does not take place conclusively and in isolation [34].

#### Mobile livelihoods

According to the study, migration can be interpreted as a livelihood strategy when opportunities fail to present themselves in Nicaragua. The concept of ‘mobile livelihoods’ [49], which highlights the embedded practice of migration in people’s strategies for making a living, captures many of the features in the study context.

A major concern for the interviewees was the struggle to make a living in Nicaragua, and the striving for a better life. Most interviewees, here referred to by fictive names, expressed that life was hard (‘la vida es dura’). Gloria (60 years, Cuatro Santos) declared in the interview that migration is often a necessity due to the difficult living conditions in rural areas. Other interviewees highlighted labour market issues as influencing people’s decisions to migrate. Cesar (31 years, León), who was a computer technician working as a taxi driver, talked about the problems of getting a qualified position and of finding jobs with decent pay in order to make ends meet. Single mothers could also find migration a necessity in order to make a living. Maribel (39 years, León), who was a nurse, had migrated both within Nicaragua and to Costa Rica to find work after having separated from her children’s father. Her story demonstrates how Nicaraguan gender structures and parenting practices place major responsibility on women for the upbringing and care of children [50], and that mothers may use migration as a strategy when assuming the role of breadwinner. Lastly, several interviewees expressed how they aspired to move forward (‘seguir adelante’) – for a better life for themselves, and for their children in the future. For Santos (33 years, León), this striving for a better life motivated three migration attempts to the US and Costa Rica. *Seguir adelante* is commonly expressed in Latin American countries, reflecting the individual’s effort of trying to escape poverty [51,52]. Similarly to previous research [53] the interviewees in this study stressed the importance of work and children’s education in order to *seguir adelante* for a better future.

#### Health concerns as embedded in mobile livelihoods

Even though very few in the survey mentioned health issues as a specific reason for future migration, the interviews showed that health concerns could influence migration decisions to a high degree, and that they often were embedded in the economic reasons that caused people to conduct mobile livelihoods.

Cesar explained in the interview that he had migrated to Costa Rica because he needed to make money in order to get glasses for himself, and because his mother was unable to go back there due to ill health. She therefore urged Cesar to go: ‘She says to me “I can’t travel, only you. You should go again, so that you can help me.”’ Cesar’s narrative points to a kind of health selectivity in migration [9], as his relatively good health situation enabled him, instead of his sick mother, to migrate. Another interviewee, Maribel, had returned to Nicaragua from working in Costa Rica in order to take care of her son, who had a drink problem, and because her mother needed assistance due to deteriorating health.

Female interviewees further mentioned having decided to migrate due to reproductive health concerns. Rosa (27 years, León), for example, had returned home to Nicaragua twice, from working in Costa Rica, when it was time to give birth to the children she was expecting.

Emotions could also influence migration decisions. Suffering and sadness, caused by the separation from family members, sometimes triggered return migration. Rosa, for example, who had spent yet another period of six months working in Costa Rica after having her third child, motivated her return to Nicaragua due to missing the children too much: ‘I suffered a lot for their sake, I wondered how they were doing, I suffered a lot … And, so, I couldn’t restrain myself, I returned.’ That Rosa says she suffers from being separated from her children can be connected to prevailing gender norms [53], and may be seen as what is expected of her to express as a mother. It can also be a highly concrete feeling influencing health. Suffering embraces many negative emotions [54] (on characterizations of emotions, see [55]). If emotions are embodied, as emotion sociologists argue [56–58], and seen as important factors influencing health, which much research shows evidence of [59–61], the emotional experience of suffering may have negative effects on both mental and physical health.

Worry and fear were other emotions that could motivate migration. Joanna (28 years, Cuatro Santos) returned from Guatemala, where she had worked for seven years, not only because she had saved up enough money to buy a house at home, which was her aim of going abroad, but also because she was afraid of the criminal gangs (*las maras*) there. Joanna’s fear is understandable, since these gangs are violent and make the population feel insecure [62,63].

Fear was highlighted in a different way in the interview with Marta (50 years, León). After separating and moving from the countryside to León with her eldest children, Marta met a new man who abused her for many years. Ten years before the interview, he suddenly left for Costa Rica and Marta was thus released from the fear and the violence. When he shortly thereafter asked if she would join him in Costa Rica, Marta declined and decided to stay in León due to the violence she had suffered. Marta’s story highlights that violence against women, which is widespread in Nicaragua [21–23], can come to an end thanks to migration.

The interview with Ana (22 years, León) also highlighted that women’s precarious situation in Nicaragua can influence migration decisions. Ana had left her birthplace, a rural community ‘in the mountains’, at the age of 15, due to a conflict within the family that arose after Ana had been sexually abused and impregnated by her father. Sexual abuse within Nicaraguan families is common; girls are seldom left alone at home, because male relatives and other men are believed to pose too great a risk to them [53]. Moreover, early pregnancy is associated with the sexual abuse of children and teenagers [20].

Lastly, the unpredictable nature conditions in Nicaragua and their effects on health could also influence people’s settlement patterns. Rosa had been forced to move twice because of natural disasters (due to a volcano eruption, and in the aftermath of Hurricane Mitch).

#### Remittances and health

In Nicaragua, remittances are an important source of income for many families [27,64]. However, as Nicaraguan emigrants often come from lower- and middle-class households, remittances tend not to go to the poorest in society, thus the development impact of remittances can be questioned [64]. This study confirms that remittances, here defined as transfers of money sent either from abroad or within the country, were often an integral part of people’s livelihoods. However, the importance of the remittances in relation to the household economy varied, from being a small supplementary part to being the main source of income. For some, remittances were what had motivated migration events in the first place. Importantly, the study showed that remittances could be significant for access to health care and medicine.

According to the survey study, 19% received remittances, which is similar to levels reported elsewhere [65]. Most (84%) used the remittances to pay for daily needs (e.g. food, clothes and housing), health costs (23%) and/or education (14%), which is consistent with previous research on the use of remittances in Nicaragua [27]. The survey also showed that over a quarter (28%) of those who had been sick (amounting to 50% of the survey respondents) received help from family and friends during times of illness, either as medicine or as money, which was used to buy medicine or to pay for private care. These findings give evidence, similarly to previous research [24–26], of a substantial lack in the Nicaraguan health sector when it comes to providing services for all, and that a functioning social network is important to access care.

The interviews further showed how important the remittances could be for people’s health needs. Juliano (24 years, Miami/León), together with his brother and father who also were US residents and lived in Miami, had on several occasions helped relatives in Nicaragua with health costs. When an uncle to Juliano was sick, they sent remittances both for medicine and to pay for care at a private clinic, because, as Juliano said, private services have ‘better medical attention’ than public ones. Juliano’s narrative shows that translocal acts of care, performed at a distance, can be crucial for access to (quality) health care and medicine in the study setting. It thus highlights the socio-economic inequities in access to health services in Nicaragua [25], and the importance of a functioning social network for receiving treatment.

The study further shows that remittances were sent not only from abroad, but also within the country. The importance of these ‘internal’ remittances – for example, in acute health situations – were seen in the interviews. Marta recounted that she had suffered from stomach cancer for which she needed surgery. The medical costs would be C$10,000, which she was unable to pay due to her economic situation. Luckily, with help from her employer, Marta managed to convince the hospital to operate on her without cost. However, she still had to pay over C$2000 for examinations, and for that she was dependent on help from others (one cousin in her birthplace and another cousin in León provided her with small amounts of money, and her employer’s foreign tenants gave her a larger sum). Marta’s narrative, like Juliano’s, illustrates the importance of a functioning social network in order to receive medical care in the study setting. Her care was managed in a translocal social network, and not as part of her social rights of citizenship, which would have entitled her to care regardless of her economic resources [9,66].

Results from binary logistic regression analysis on the survey data showed a strong positive association between those who received remittances and those who received help when they were sick (see [1], p. 193). Yet the regressions did not show any associations between remittance-receiving and health status; for example, self-rated physical health. Hence, in general, health status did not seem to have an effect on remittance-receiving. But, in the event of illness, those who usually received remittances were more likely also to do so during the illness period. This is an important finding, since we know that remittances are often used to pay for both health care and medicine due to Nicaragua’s non-inclusive health care system. Those who receive remittances during the illness period may thus have better access to health care than others, which may improve their health situation.

The regressions also showed that remittance-receivers were more likely to be older and either non-skilled workers, housewives, students or non-economically active. These groups perhaps receive more remittances because of a disadvantaged economic situation. Moreover, the results confirmed previous findings [30] that remittances are often sent from the US (see [1], pp. 188–193, for more details on the analysis of remittance-receivers).

### Health on the move

Migration is commonly regarded as a stressful life event [67] that can have diverse effects on health and access to health care and medicine. The qualitative findings of this study show that the migrants’ health was affected not only during the journey and during the life in the new place, but also at the return home.

#### The journey: crossing borders

According to the interviews, the international migrants were the most vulnerable during the journey. Immigration status was central for the stress and risks the border crossings entailed. Borders are often dangerous settings, and migrants can suffer both physically and mentally during the journey and at the border crossing [68–74]. Santos, who had tried repeatedly to get to the US and Costa Rica, emphasized in the interview that his migrations had entailed huge risks and painful situations, always being at the mercy of the smugglers (*coyotes*) who made the passage possible, and worrying about the safety of his life or for being kidnapped. On one occasion Santos travelled through Mexico on the infamous cargo train known as ‘The Beast’ (*La Bestia*), risking severe injury and even death due to falling off the train. Gangs also patrol this passage route, exposing the migrants to violence, assault, extortion and kidnapping [73,74]. On the road, Santos had not only suffered personally, from hunger, thirst, maltreatment and humiliation from border police, amongst other things, but he had also seen one of his friends die, and witnessed female migrants being sexually abused. Santos’ narrative highlights the vulnerability and suffering connected to irregular migration, and also shows how psychologically trying the ‘animalization’ of border crossers [68] can be for migrants, as he was deeply hurt by being treated ‘as a dog’ by the border police.

The interviews further showed that those who crossed the American border irregularly were subjected to the worst dangers, but that it was also experienced as stressful to cross the Costa Rican border without documents. Moreover, the migrants who could cross borders legally were in a much less vulnerable situation, and usually experienced less stress, which indicates that social differentiations (in this case, legal status) matter for the health consequences of border crossings. Furthermore, even though the international migrants were exposed to the most perilous situations, the internal migrants (e.g. Ana) could also go through difficulties and dangers on the way to the destination, which I believe is important to acknowledge.

#### Life in the new place

At the destination the migrants’ health was affected in both positive and negative ways. Some interviewees’ social milieu changed for the better in relation to migration events (as in Ana’s case, an end to abuse and conflict, and gaining new, supportive relationships). Some learnt new skills after moving (like Juliano, who received training as a painter in the US), which is positive as human capital is a well-known determinant of health [66]. Still, many migrants experienced the move to a new place as highly stressful due to the spatial, sociocultural and environmental disruptions that took place in the individual’s life space. Leaving behind familiar settings and adapting to a new place and culture has been shown to be stressful in many studies [9], due to the processes of ‘estrangement’ [75] and ‘cultural bereavement’ [67]. In the interviews this was expressed as bodily discomfort (e.g. health problems due to climate change) and feelings of loss of familiar land and culture, often manifested as homesickness.

Several interviewees who had migrated internationally talked about being subject to discrimination and maltreatment, and they also experienced a fear of violence. Maribel recounted that she had endured many xenophobic comments when she was living in Costa Rica, which was stressful and affected her a lot emotionally. Because of a ‘fear of pollution’ [76], migrants are often viewed as a threat and consequently ‘othered’ because of their ‘different’ appearance [75,77]. The ‘othering’ of Nicaraguan migrants is very strong in the case of Costa Rica, where Nicaraguans are outlined as a ‘communist threat’, and associated with dark skin, poverty, crimes and disease [77]. The consequences of ‘othering’ (xenophobia and racism) may produce stress and other negative health consequences for migrants [9,78].

The interview findings further show how migrant workers – and especially if they are undocumented – are exposed to structural discrimination in the labour market as well as a lawless situation that can produce negative effects on health. The labour system in place today is characterized by a high degree of precariousness [79]. At work, the physical being is intimately connected to socio-economic relations, often characterized by unequal power relations [80]. Today’s global, capitalist labour system is not only hierarchically organized and geographically differentiated, it is also racialized and gendered, and exploits workers based on their social position [81,82]. Migrant workers are in a particularly vulnerable situation, as they are newcomers who are less familiar with the labour market, and often also have fewer rights [83]. Precarious work may have detrimental effects on health [84], and the lawless situation many undocumented workers are in also often produces negative effects on health and access to health care [85]. It is important to acknowledge that some interviewees in the study did not experience the negative sides of migrant work. These migrants often worked ‘legally’ (e.g. Juliano in Miami), or in jobs that were ‘invisible’ from public view (e.g. Rosa in Costa Rica, who was able to stay overnight at her workplaces). Another aspect that lessened the degree of vulnerability and suffering was skin colour. Rosa said in the interview that she believed that part of the reason for her good experiences from working in Costa Rica was related to her ‘whiteness’ – that is, her pale skin colour made her look more Costa Rican than Nicaraguan, which meant that she did not have to endure as much xenophobia as other Nicaraguans. This shows that social position – for example, legal status, gender and ethnicity/‘race’ – influence the degree of precariousness and vulnerability for the migrant worker [83].

The interviews also showed that the migrants’ access to health care often changed, for the better or for the worse. Internal migrants often gained better access when moving to urban areas, since access to health care in rural areas was much more limited. This was highlighted, for example, in the interview with Ana, who described it as very easy to access health services in León compared to in her home village, where the closest health care centre lay in the nearest village, two hours away by foot, and the nearest hospital was a whole day’s walk away. Ana attributed the deaths of several of her siblings to the long distance to the hospital, as well as that the family was unable to afford medicine. She further pointed out the necessity of help within the community for accessing health care. Ana’s story illustrates the geographical inequities in the access to and use of health care services in Nicaragua [25], and confirms the proposition that the distance to health care services impacts the use of health care [9]. It also highlights that, when the distribution of services is uneven, social networks become crucial for obtaining care.

In contrast to the rural–urban migrants, the international migrants (and particularly the undocumented) often faced limitations to their access to health care after moving. Based on their nationality and legal status, both Maribel and Cesar, for example, were discriminated against in relation to employers and health care services in Costa Rica, and therefore had to pay cash in order to get care and medicine during periods of illness.

Hence, several factors in the migrants’ lives at the destination influenced the migrants’ health. On the positive side, some migrants experienced improvements in their social milieu, learnt new skills and improved their access to health care (i.e. rural–urban migrants). On the negative side, other migrants experienced feelings of estrangement and cultural bereavement, as well as the consequences of ‘othering’. Moreover, particularly the undocumented international migrants were in a precarious and vulnerable situation, affecting their working and living conditions, as well as access to health care and medicine. Social position was key to this process, affecting the degree of vulnerability and suffering.

#### Returning ‘home’

The interviewees depicted the process of returning home very differently depending on the circumstances surrounding migration. Rosa, for example, had always been very happy to return home from Costa Rica, because it meant she could reunite with her children. Joanna, however, experienced ambivalent feelings – that is, a mix of both positive and negative emotions – in relation to her return from Guatemala to Cuatro Santos. On the one hand, she was happy to have returned because she had experienced great sadness being away from her home community, as well as a fear of violence because of the gangs in Guatemala. She was also very happy since she now lived in a house of her own, which she and her husband had bought with the money they had made in Guatemala. On the other hand, Joanna said that she missed working and making money of her own, which was not possible in Cuatro Santos because of lacking job opportunities. After returning she had become more dependent on her husband, who sent money for the family’s sustenance: a step back in terms of gender equality, one might say.

Finally, for Santos, the return to León was primarily characterized by negative emotions. In his mind, his migration attempts had been ‘unsuccessful’, since he had on all occasions been caught by the border police and deported to Nicaragua. Santos felt sad and ashamed at his ‘failure’ when he returned to Nicaragua, and also alienated from his family due to the separation. Stress that causes negative emotional experiences, such as sadness and shame in Santos’ case, can affect not only mental but also physical health through the onset of biological processes that in turn affect disease risk and mortality [86,87]. Santos’ mental health was clearly affected; in the interview he said that he had thought about ending his own life, and that some of his friends after ‘unsuccessful’ migrations had in fact committed suicide, which according to previous Nicaraguan studies can be a risk factor influencing others to attempt suicide [88–90]. Coping strategies – that is, the attempts to manage stressful situations that feel difficult to handle – can nevertheless be enacted when encountering stress [91–94], and hinder the most tragic effects of difficult life experiences. Research shows that coping strategies can be more or less beneficial for health. One way of coping for Santos was to share his experiences with others in talk (as in the interview) and writing (he had written a short story about his experiences). Storytelling may have a therapeutic effect and be beneficial for health [60,95,96].

The interviews thus showed that the process of return can stir up both positive and negative emotions; emotions that, according to mind/body medicine [8] and the theories on emotions and health, may influence both physical and mental health [56–61,86,97]. Return migrants’ health is an under-researched issue, determined by a ‘cumulative exposure’ to risks and behaviour during the entire migration process; that is, by conditions and events before, during and after the actual migration [98]. The findings in this study confirm this, in that the circumstances surrounding migration events seemed highly influential in how the return was experienced by the migrant, and in the consequences on health.

### Health and coping in divided families

Due to migration, social relations are spatially stretched out [99], causing transformations in family relations. The ‘translocal social lives’ that are produced in this process can create stress for both migrants and left-behind family members [100–102]. Many of the interviewees in this study witnessed changes in family relations: from dissolutions after having been abandoned, causing suffering and resentment on behalf of the staying spouse, to changes in parent–child relations due to an absent mother or father, causing pain and difficulties for both parents and children involved. The study findings highlight the problems associated with ‘transnational parenthood’ [103] – for example, the changes in parent–child relations, and the difficulties for left-behind mothers in taking on additional responsibilities in terms of discipline and decision-making, which may be experienced as overwhelming. The study also illustrates the dilemma associated with transnational motherhood. Even though shared mothering is commonly practised in Nicaragua, whereby female relatives other than the mother partake in the upbringing of children [104], difficulties can still arise due to the separation from children [105]. Migrating mothers often continue to be responsible for the emotional care of their children, and frequently express emotional distress and guilt due to their absence, even though communication is maintained [106–108]. If they are confident of their children’s well-being it may, however, be easier to focus on breadwinning, which then may be considered a valid form of caregiving [105].

#### Emotional and physical health impacts of separation

The interviews clearly showed how emotionally affected the interviewees were by migration events and the translocal lives they were leading. In line with previous research [109], migration seemed to be surrounded by emotional contradictions. Even though some communicated positive emotions – for example, when destructive relationships were brought to an end thanks to migration events – most interviewees expressed that the separation from their families and home communities was hard on them. For some, it entailed high psychological costs. The experience of negative emotions such as sadness, suffering and worry, which the interviewees expressed, can have negative effects on health, according to the literature [56–61,86,97]. Rosa, who at the time of the interview was working outside León while her children stayed with her mother about half-a-day’s trip from Rosa’s workplace, expressed how during the many years of migrant work she had suffered because of the separation from her children. Even though Rosa’s separation from her children was justified by the need to make a living, the advantages of migration (i.e. breadwinning) did not seem to take away the pain of separation for Rosa. Furthermore, some interviewees expressed both positive and negative feelings simultaneously. Juliano, for example, seemed to experience an ambivalence about migration due to the conflict between working in order to make money and get what they needed and wanted in life, and enduring family separation due to migration:

It’s good to be there [in the US], because you can help people. […] What it affects the most, is the family … Luxury and things like that there, it’s not worth it … The truth is, if you’re not together with the ones you want to be with and share it … […] Everyone wants to go to another country, maybe for the [economic] conditions, but when you’re there, it’s another thing – then the money doesn’t matter any longer, what interests you is the family … to be here. […] As I say, there are advantages and disadvantages.

The interviews further showed that the migration-induced changes in parent–child relations could have consequences on child health. Rosa recounted that her youngest son had suffered an ear infection when she had worked in Costa Rica the last time, which went untreated and resulted in long-term damage to his hearing. The boy had also been depressed, and he suffered from heart problems. In Rosa’s mind, it was the separation from her and the boy’s father (who had abandoned them) that caused the boy’s health problems, but that they were aggravated by her relatives’ neglect and maltreatment of the boy. She felt disheartened when she returned from working for the sake of her children and found the boy in this poor condition. According to Juliano’s wife Cindy (24 years, León), Juliano’s absence had also been very hard on their son; the boy had stopped eating normally, he slept poorly and he had been very sad. With help from the school psychologist, and through adaptation over time, the boy’s situation had nevertheless improved. Left-behind children are often negatively affected when parents migrate. Feelings of abandonment, reproach and longing, both during the time when the parents are gone and when/if reunification takes place, as well as a feeling of loss are common, which can have serious effects on the children’s well-being [110]. It is therefore important to help children to cope with parents’ absence [100,103,111].

In the qualitative study many thus experienced the separation from family members as psychologically trying, sometimes also having implications on the left-behinds’ health. Regression analysis of the survey data also showed an association between migration events and physical health – that is, family members of out-migrants (‘Left-behinds’) more seldom rated their physical health as good, also when poverty, sex and age were controlled for (see [1], p. 257), compared to individuals residing in non-migrant households (‘Non-movers’). This finding can have several alternative explanations. The left-behinds may live in a stressful situation for different reasons. Health problems in the family may trigger migration, and, due to the healthy migrant selection mechanisms, the healthy individuals (with the capacity to leave) migrate, leaving those with health problems behind. Whatever may be the reason, it is obviously more likely for family members of migrants to rate their health as bad. Further analysis showed that people who had undocumented relatives were more likely to rate their physical health as bad (see [1], p. 258). (However, it is possible that being a family member to an undocumented migrant is merely a proxy for being poor, since the analysis indicates a correlation between family members to undocumented migrants and poverty; the poor were less likely to rate their health as good). This can be seen in light of the findings from the interview study that reveal the negative effects of being an undocumented migrant, and the stress that family members left behind may experience because of this.

#### Coping with separation and caring at a distance

Families who are divided over time and space often maintain a sense of familyhood, through different strategies [112]. According to the results from the survey study, contact with dispersed family members was very common within the study population: 93% had contact with relatives living abroad, most often by telephone (84%). The qualitative study showed that the interviewees employed several different strategies for coping with the separation from their loved ones, and for expressing care. Investing in relationships and maintaining good communication was very important. The efforts of creating a ‘virtual co-presence’ [113] in order to maintain relationships did not, however, come without cost and sacrifice. Juliano phoned home to his wife Cindy and their son from Miami daily, and even more often at weekends, even though the phone calls were costly. The frequent communication made it possible for them to maintain their relationship, Cindy said, and Juliano could stay close to his son. Maribel also called her children on the phone regularly when she lived in Costa Rica. She reasoned that it was her obligation as a mother to make the calls, even if sometimes she could not afford it and it meant going without eating.

Juliano’s annual visit to Nicaragua was furthermore very important for Cindy and Juliano to keep together as a family. They tried to make the most (*aprovechar*) of these visits, which can be seen as a way of coping.

To have a positive attitude, to keep the faith and to make joint plans were other ways of coping with separation within families. Cindy and Juliano were waiting and hoping for the day the US residency application for Cindy and their son was approved, so that they could move to Miami. Juliano and Cindy’s plan for reunification, and their goal to work for a couple of years in the US and then return to León, as well as taking the necessary steps to make it come true, can be seen as strategies of coping, with both the difficult economic situation in Nicaragua and the separation from each other.

For some, the socio-economic gains of migration made the separation from loved ones slightly easier to cope with. Juliano said, in relation to the money he was sending to family and friends in Nicaragua, that it ‘felt good’ to have the possibility to help others. Maribel and Rosa also made an effort to see the economic advantages of their migration in order to ease the separation from their children. They suffered but stressed that they needed to make money to support themselves and their children. The focus on the positive aspect of migration (the breadwinning) thus ‘justified’ their absence, which can be seen as a way of coping with the distress they experienced.

Working hard and sending remittances can be seen as acts of love, of caring at a distance, as previous studies also argue [114–116]. Although some of the migrants in the study suffered from precarious working and living conditions, many said that the improved economic situation they gained through the migrant work made the tough times worth it. The emotional pain of separation was nevertheless often a constant agony. As other studies [101] show, stress and psychological distress resulting from familial separation can be common, regardless of the strategies employed to sustain relations.

### Conclusions

The overall conclusions of the study can be summarized in these four points:

- In Nicaragua, the social rights of citizenship are limited due to historical and present-day structural socio-economic relations at different scales. This affects the labour market, the health care system and the educational sector, necessitating people’s mobile livelihoods and the need for translocal social support (e.g. remittances).
- Migration can have both positive and negative effects for migrants and their family members. For example, rural–urban migrants can in some respects sometimes be favoured by moving. However, this study points to overwhelmingly negative consequences (e.g. vulnerability, precariousness, emotional costs, etc.), but it depends on the social position of the migrant. Acknowledgment of social differences (e.g. gender, ethnicity/’race’, age, immigration status, etc.) is therefore key for the enactment of the migration–health nexus. Moreover, geographical distance is another important factor to take into account in the analysis of migration–health relations.
- It is important to acknowledge the role and importance of health issues in migration studies, as these can be embedded in people’s mobile livelihoods (e.g. migration motives).
- The study points to the benefits of mixing qualitative and quantitative methods for understanding the complex relation between migration and health.

## Paper context

This study augments limited and outdated research on migration and health in Latin America through comprehensively analysing the migration–health nexus in Nicaragua, which is a dynamic and complex process connected to broader socio-economic factors. Findings show that limits in the social rights of citizenship produce mobile livelihoods, which have some positive, but predominantly negative, health consequences for migrants and their families. However, geographical distance and social differences are key to the outcome.

## References

[1] Gustafsson C. *‘“For a better life…”* A study on migration and health in Nicaragua’. PhD Dissertation Vol. 2 Department of Geography and Economic History, Umeå University Serie: GERUM 2014. 10.1080/16549716.2018.1428467 PMC6041780 29975179

[2] McKay L, Macintyre S, Ellaway A Migration and Health: A review of the international literature. Glasgow: Medical Research Council, Social and Public Health Sciences Unit, University of Glasgow; 2003.

[3] Schærström A, Rämgård M, Löfman O Hälsans och ohälsans landskap: från medicinsk geografi till hälsogeografi [Landscapes of health and disease: from medical geography to health geography]. 1. uppl ed. Lund: Studentlitteratur AB; 2011.

[4] Cabieses B, Tunstall H, Pickett KE, et al Changing patterns of migration in Latin America: how can research develop intelligence for public health? Pan Am J Public Health. 2013;34:68. 24006023

[5] Moon G Health geography In: Kitchin R, Thrift N, editors. International encyclopedia of human geography. Amsterdam/Boston: Elsevier Ltd; 2009.

[6] White P Biopsychosocial medicine: an integrated approach to understanding illness. 1st ed. White P, editor. Oxford: Oxford University Press; 2005.

[7] Parr H, Butler R New geographies of illness, impairment and disability In: Butler R, Parr H, editors. Mind and body spaces: geographies of illness, impairment and disability. London: Routledge; 1999.

[8] Dreher H Mind-body unity: a new vision for mind-body science and medicine. Baltimore: Johns Hopkins University Press; 2004.

[9] Gatrell AC, Elliott SJ Geographies of health: an introduction. Vol. 2 Singapore: Wiley-Blackwell; 2009.

[10] Brickell K, Datta A, editors. Translocal geographies: spaces, places, connections. Farnham: Ashgate; 2011.

[11] Haour-Knipe M Context and perspectives: who migrates and what are the risks? In: Thomas F, Gideon J, editors. Migration, health and inequality. London: Zed Books; 2013.

[12] Zimmerman C, Kiss L, Hossain M Migration and health: a framework for 21st century policy-making. PLoS Med. 2011;8:e1001034. 21629681 10.1371/journal.pmed.1001034 PMC3101201

[13] Gutiérrez C, Paci P, Ranzani M Making work pay in Nicaragua: employment, growth, and poverty reduction. Washington (DC): World Bank; 2008.

[14] UNDP Human Development Report 2013. The Rise of the South: Human Progress in a Diverse World. New York (NY): United Nations Development Programme; 2013.

[15] CEPAL Nicaragua: evolución economica durante 2010 y perspectivas para 2011 [Nicaragua: economic development during 2010 and prospects for 2011]. México: United Nations Economic Commission for Latin America and the Caribbean (UN-CEPAL); 2011.

[16] Pozzoli D, Ranzani M Participation and sector selection in nicaragua. Working Paper 09-8. Aarhus (Denmark): Aarhus School of Business, Department of Economics, Aarhus University.

[17] INIDE Encuesta de hogares sobre medición del nivel de vida 2009. Principales resultados: pobreza, consumo, ingreso [Household survey on living standards 2009. Main results: poverty, consumtion, income]. Managua: National Institute for Information and Development; 2011.

[18] UNdata [Internet] Division. Accessed 2014 2 13 Available from: http://data.un.org/Explorer.aspx?d=WDI.

[19] Pérez W Millennium Development Goals in Nicaragua: analysing progress, social inequalities, and community actions. PhD Dissertation Uppsala (Sweden): Faculty of Medicine, Uppsala University; 2012.

[20] Zelaya Blandón E Adolescent pregnancies in Nicaragua: The importance of education. PhD Dissertation Umeå (Sweden): Epidemiology, Department of Public Health and Clinical Medicine, Umeå University; 1999.

[21] Valladares Cardoza E Partner violence during pregnancy, psychosocial factors and child outcomes in Nicaragua. PhD Dissertation Umeå (Sweden):Epidemiology, Department of Public Health and Clinical Medicine, Umeå University; 2005.

[22] Ellsberg MC Candies in hell: Research and action on domestic violence against women in Nicaragua. PhD Dissertation Epidemiology, Department of Public Health and Clinical Medicine, Umeå University; 2000.

[23] INIDE The Nicaraguan demographic and health survey 2006/2007. Managua: National Institute for Information and Development; 2008.

[24] PAHO Health systems profile in nicaragua: monitoring and analyzing health systems change/reform. 3 ed. Washington (DC): Pan American Health Organization; 2009.

[25] Angel-Urdinola DF, Cortez R, Tanabe K Equity, access to health care services and expenditures on health in nicaragua. Washington(DC): International Band for Reconstructions and Development/The World Bank; 2008.

[26] Sequeira M, Espinoza H, Amador JJ, et al The Nicaragua Health System: an overview of critical challenges and opportunities. Seattle: Program for Appropriate Technology in Health; 2011.

[27] Morales A, Castro C Redes Transfronterizas: sociedad, empleo y migración entre Nicaragua y Costa Rica [Transborder networks: society, employment and migration between Nicaragua and Costa Rica]. San José: Latin American Faculty of Social Sciences (FLACSO); 2002.

[28] IOM Perfil Migratorio de Nicaragua 2012 [Migration Profile for Nicaragua 2012]. Managua: International Organization for Migration; 2013.

[29] Vivas Vivachica EA Migración interna en Nicaragua: descripción actualizada e implicancias de política, con énfasis en el flujo rural-urbano [Internal migration in Nicaragua: updated description and political implications, with focus on rural-urban movements]. Santiago de Chile: Latin American and Caribbean Centre of Demography (CELADE), Population Division of CEPAL, UNFPA; 2007.

[30] UNDP Human development report 2009. Overcoming barriers: Human mobility and development. New York, NY: United Nations Development Programme; 2009.

[31] Watkins D, Gioia D Mixed methods research. New York, NY: Oxford University Press; 2015.

[32] Teddlie C, Tashakkori A Foundations of mixed methods research: integrating quantitative and qualitative approaches in the social and behavioral sciences. Los Angeles (CA): SAGE; 2009.

[33] Charmaz K Grounded theory: objectivist and constructivist methods In: Denzin NYL, editors. Strategies of qualitative inquiry. 2 ed. Thousand Oaks (CA): SAGE; 2003.

[34] Halfacree KH, Boyle PJ The challenge facing migration research: the case for a biographical approach. Prog Hum Geogr. 1993;17:333. 12286809 10.1177/030913259301700303

[35] Skeldon R The challenge facing migration research - a case for greater awareness. Prog Hum Geogr. 1995;19:91–14. 12292946 10.1177/030913259501900106

[36] Findlay AM, Li FLN An auto-biographical approach to understanding migration: the case of Hong Kong emigrants. Area. 1997;29:34–44.

[37] Peña R, Pérez W, Meléndez M, et al The Nicaraguan Health and Demographic Surveillance site, HDSS- León: a platform for public health research. Scand J Public Health. 2008;36:318–325. 18519303 10.1177/1403494807085357

[38] Peña R, Pérez W, Meléndez M, et al Reporte de Línea de Base del Sistema de Vigilancia en Demografía y Salud, León, Nicaragua, 2002 [Report from the Health and Demographic Surveillance System, León, Nicaragua, 2002]. Unpublished report. León, Nicaragua: Centre for Demographic and Health Research (CIDS) and Umeå University; 2005.

[39] Zelaya Blandón E, Peña R, Betancourth S, et al Actualización de Línea de Base. Segundo Reporte del Sistema de Vigilancia Demográfica y de Salud de los Municipios San Juan de Cinco Pinos, Santo Tomas, San Francisco y San Pedro del Norte [Actualization of the baseline. Second report from the Health and Demographic Surveillance System in the municipalities San Juan de Cinco Pinos, Santo Tomas, San Francisco and San Pedro del Norte]. Unpublished report. León, Nicaragua: Centre for Demographic and Health Research (CIDS) and APRODESE; 2008.

[40] Faist T The Volume and Dynamics of International Migration and Transnational Social Spaces. Oxford: Oxford University Press; 2000.

[41] Glick Schiller N, Faist T, editors. Migration, Development and Transnationalization. A critical stance. New York: Berghahn Books; 2010.

[42] Tollefsen Altamirano A Seasons of migrations to the north. A study of biographies and narrative identities in US-mexican and swedish-chilean return movements. PhD Dissertation. Umeå (Sweden):Department of Social and Economic Geography, Umeå University Serie: GERUM Kulturgeografi 2000:3.

[43] Levitt P, Schiller NG Conceptualizing simultaneity: a transnational social field perspective on society 1. Int Migration Rev. 2004;38:1002–1039.

[44] Vertovec S Transnationalism. London: Routledge; 2009.

[45] Douglas SM, Mariano S Patterns of U.S. Migration from Mexico, the caribbean, and central America. Migraciones Internacionales. 2003;2:5–39.

[46] De Jong GF, Gardner RW, editors. Migration decision making: multidisciplinary approaches to microlevel studies in developed and developing countries. New York (NY): Pergamon; 1981.

[47] Robinson V, editor. Geography and migration. Cheltenham: Edward Elgar Publishing Company; 1996.

[48] Skeldon R Population mobility in developing countries: a reinterpretation. London: Belhaven; 1990.

[49] Olwig KF, Sørensen NN Mobile livelihoods: making a living in the world In: Sørensen NN, Olwig KF, editors. Work and migration: life and livelihoods in a globalizing world. London: Routledge; 2002.

[50] Franzoni JM, Voorend K Who cares in nicaragua? A care regime in an exclusionary social policy context. Dev Change. 2011;42:995–1022. 22164883 10.1111/j.1467-7660.2011.01719.x

[51] Steel G, Winters N, Sosa C Mobility, translocal development and the shaping of development corridors in (semi-) rural Nicaragua. Int Dev Plann Rev. 2011;33:409–428.

[52] Leinaweaver JB Improving oneself: young people getting ahead in the peruvian andes. Lat Am Perspect. 2008;35:60–78.

[53] Johansson A La mujer sufrida – the suffering woman: narratives on femininity among women in a nicaraguan barrio. Vol. 70 Monograph from the Department of Sociology, Göteborg University. Gothenburg: Göteborg University; 1999.

[54] Wilkinson I Suffering: A Sociological Introduction. Cambridge: Polity Press; 2005.

[55] Parrott GW, editor. Emotions in social psychology: essential readings. New York: Psychology Press; 2001.

[56] Williams SJ, Bendelow G Emotions, health and illness: the ‘missing link’ in medical sociology? In: James V, Gabe J, editors. Health and the sociology of emotions. Oxford: Blackwell Publishers; 1996.

[57] Lupton D Going with the flow: some central discourses in conceptualising and articulating the embodiement of emotional states In: Nettleton S, Watson J, editors. The body in everyday life. London: Routledge; 1998.

[58] Barbalet J, editor. Emotions and sociology. Oxford: Blackwell Publishing; 2002.

[59] Folkman S The oxford handbook of stress, health and coping. New York (NY): Oxford University Press; 2011.

[60] Pennebaker JW Emotion, disclosure, and health: an overview In: Pennebaker J, editor. Emotion, disclosure, and health. Washington (DC): American Psychological Association; 1995.

[61] Lazarus RS Stress and emotion: a new synthesis. New York (NY): Springer Publishing Company; 2006.

[62] Aguilar J, Carranza M Las Maras y Pandillas como Actores Ilegales de la Región [Las Maras and Gangs as Illegal Actors in the Region]. Informe Estado de la Región 2008 [Report on the State of the Region]. Unpublished Report. San Salvador; 2008.

[63] Cruz JM El barrio transnacional: las maras centroamericanas como red [The transnational neighbourhood: the Central American gangs as network] In: Pisani, editor. Redes transnacionales en la Cuenca de los Huracanes Un aporte a los estudios interamericanos [Transnational networks in the Hurricane Basin A report on Interamerican studies]. México: Instituto Autónomo de México; 2005.

[64] Jennings A, Clarke M The development impact of remittances to Nicaragua. Dev Pract. 2005;15:685–691.

[65] Fajnzylber P, López HJ Close to home: the development impact of remittances in latin America. Washington (DC): International Bank for Reconstruction and Development/World Bank; 2007.

[66] Turner B The new medical sociology: social forms of health and illness. New York (NY): W.W. Norton & Company; 2004.

[67] Helman CG Culture, health and illness. Vol. 5 London: Hodder Arnold; 2007.

[68] Khosravi S ‘Illegal’ Traveller: an auto-ethnography of borders. Basingstoke: Palgrave Macmillan; 2011.

[69] Eschbach K, Hagan J, Rodriguez N, et al Death at the border. Int Migr Rev. 1999;33:430–454. 12319738

[70] Holmes SM ‘Is it worth risking your life?’: ethnography, risk and death on the U.S.–mexico border. Soc Sci Med. 2013;99:153–161. 24120251 10.1016/j.socscimed.2013.05.029

[71] Slack J, Whiteford S Violence and migration on the Arizona-Sonora border. Hum Organ. 2011;70:11–21.

[72] Binational Migration Institute A continued humanitarian crisis at the border: undocumented border crosser deaths recorded by the pima county office of the medical examiner, 1990-2012. Tucson: University of Arizona, Department of Mexican American Studies; 2013 6.

[73] Cornelius WA Death at the border: efficacy and unintended consequences of US Immigration control policy. Popul Dev Rev. 2001;27:661–685.

[74] Orraca Romano PP Corona villavicencio FdJ. Risk of death and aggressions encountered while illegally crossing the U.S.- Mexico border. Migraciones Internacionales. 2014;7:9–41.

[75] Ahmed S Strange encounters: embodied others in post-coloniality. London: Routledge; 2000.

[76] Douglas M Purity and danger: an analysis of concepts of pollution and taboo. London: Routledge; 1966.

[77] Sandoval-García C Threatening Others: nicaraguans and the formation of national identities in Costa Rica. Ohio: Center for International Studies, Ohio University; 2004.

[78] Williams DR, Neighbors HW, Jackson JS Racial/ethnic discrimination and health: findings from community studies. Am J Public Health. 2003;93:200–208. 12554570 10.2105/ajph.93.2.200 PMC1447717

[79] Standing G The Precariat: the new dangerous class. Vol. 2 London: Bloomsbury; 2014.

[80] Wolkowitz C Bodies at work. London: SAGE; 2006.

[81] Bonacich E, Alimahomed S, Wilson JB The racialization of global labor. Am Behav Scientist. 2008;52:342–355.

[82] Lugones M Heterosexualism and the colonial/modern gender system. Hypatia. 2007;22:186–209.

[83] McDowell L, Batnitzky A, Dyer S Precarious work and economic migration: emerging immigrant divisions of labour in greater London’s service sector. Int J Urban Reg Res. 2009;33:3–25.

[84] Tompa E, Scott-Marshall H, Dolinschi R, et al Precarious employment experiences and their health consequences: towards a theoretical framework. Work. 2007;28:209–224. 17429147

[85] Brabant Z, Raynault M-F Health situation of migrants with precarious status: review of the literature and implications for the Canadian Context— part A. Soc Work Public Health. 2012;27:330–344. 22657147 10.1080/19371918.2011.592076 PMC3438487

[86] Greco M, Stenner P Emotions: a social science reader. New York (NY): Routledge; 2008.

[87] Weiss GL, Lonnquist LE The sociology of health, healing, and illness. 3 ed. New Jersey: Prentice Hall; 2000.

[88] Obando Medina C When no-one notices… Studies on suicidal expression among young people in Nicaragua. PhD Dissertation Umeå (Sweden):Department of Clinical Sciences, Division of Psychiatry, Umeå University; 2011.

[89] Herrera Rodríguez A Heaven can wait: Studies on suicidal behaviour among young people in Nicaragua. PhD Dissertation Umeå (Sweden):Department of Clinical Sciences, Division of Psychiatry, Umeå University; 2006.

[90] Caldera Aburto TJ Mental health in Nicaragua: with special reference to psychological trauma and suicidal behaviour. PhD Dissertation Umeå (Sweden):Department of Clinical Sciences, Division of Psychiatry, Umeå University; 2004.

[91] Thoits PA Stress and health: major findings and policy implications. J Health Soc Behav. 2010;51:S41–S53. 20943582 10.1177/0022146510383499

[92] Folkman S, Moskowitz JT Coping: pitfalls and promise. Annu Rev Psychol. 2004;55:745–774. 14744233 10.1146/annurev.psych.55.090902.141456

[93] Lazarus RS, Folkman S Stress, appraisal, and coping. New York (NY): Springer Publishing Company; 1984.

[94] Antonovsky A Health, stress, and coping: new perspectives on mental and physical well-being. San Franscisco (CA): Jossey-Bass Inc; 1979.

[95] Pennebaker JW, Seagal JD Forming a story: the health benefits of narrative. J Clin Psychol. 1999;55:1243–1254. 11045774 10.1002/(SICI)1097-4679(199910)55:10<1243::AID-JCLP6>3.0.CO;2-N

[96] Rosenthal G The healing effects of storytelling: on the conditions of curative storytelling in the context of research and counseling. Qual Inq. 2003;9:915–933.

[97] Vitetta L, Anton B, Cortizo F, et al Mind-Body medicine: stress and its impact on overall health and longevity. Ann N Y Acad Sci. 2005;1057:492–505. 16399915 10.1196/annals.1322.038

[98] Davies AA, Borland RM, Blake C, et al The dynamics of health and return migration. PLoS Med. 2011;8:e1001046. 21738448 10.1371/journal.pmed.1001046 PMC3124523

[99] Massey D For Space. London: SAGE; 2005.

[100] Silver A Families across borders: the emotional impacts of migration on origin families. Int Migration. 2014;52:194–220.

[101] Schmalzbauer L Searching for wages and mothering from afar: the case of honduran transnational families. J Marriage Fam. 2004;66:1317–1331.

[102] Pribilsky J ‘Aprendemos A Convivir’: conjugal Relations, Co‐parenting, and Family Life Among Ecuadorian Transnational Migrants in New York and The Ecuadorian Andes. Global Networks. 2004;4:313–334.

[103] Carling J, Menjívar C, Schmalzbauer L Central Themes in the Study of Transnational Parenthood. J Ethn Migr Stud. 2012;38:191–217.

[104] Winters N Responsibility, Mobility, and Power: translocal Carework Negotiations of Nicaraguan Families. Int Migration Rev. 2014;48:415–441.

[105] Nicholson M Without their children: rethinking motherhood among transnational migrant women. Social Text. 2006;24:13–33.

[106] Parreñas RS Mothering from a distance: emotions, gender, and intergenerational relations in Filipino transnational families. Feminist Stud. 2001;27:361–390.

[107] Parreñas R Long distance intimacy: class, gender and intergenerational relations between mothers and children in Filipino transnational families. Global Networks. 2005;5:317–336.

[108] Hondagneu-Sotelo P, Avila E I’m here, but I’m there: the meanings of Latina transnational motherhood. Gend Soc. 1997;11:548–571.

[109] Svašek M Who cares? Families and feelings in movement. J Intercultural Stud. 2008;29:213–230.

[110] Suárez‐Orozco C, Todorova ILG, Louie J Making up for lost time: the experience of separation and reunification among immigrant families. Fam Process. 2002;41:625–643. 12613121 10.1111/j.1545-5300.2002.00625.x

[111] Graham E, Jordan LP, Yeoh BSA, et al Transnational families and the family nexus: perspectives of indonesian and filipino children left behind by migrant parent(s). Environ Plann A. 2012;44:793–815. 10.1068/a4445 PMC3836409 24273371

[112] Baldassar L, Merla L Introduction: transnational family caregiving through the lens of circulation In: Baldassar L, Merla L, editors. Transnational families, migration and the circulation of care: understanding mobility and absence in family life. routledge research in transnationalism. New York: Routledge; 2013.

[113] Baldassar L Missing kin and longing to be together: emotions and the construction of co-presence in transnational relationships. J Intercultural Stud. 2008;29:247–266.

[114] Leifsen E, Tymczuk A Care at a distance: ukrainian and ecuadorian transnational parenthood from Spain. J Ethn Migr Stud. 2012;38:219–236.

[115] McKenzie S, Menjívar C The meanings of migration, remittances and gifts: views of Honduran women who stay. Global Networks. 2011;11:63–81.

[116] Fouratt C Por El Amor Y La Tierra: las Inversiones Emocionales De Los Migrantes Nicaragüenses [For love and the nation: the emotional investments of nicaraguan migrants]. Anuario De Estudios Centroamericanos, Universidad De Costa Rica. 2012;38:193–212.

